# Sleep apnea pathophysiology

**DOI:** 10.1007/s11325-023-02783-7

**Published:** 2023-03-28

**Authors:** Giovanni Andrisani, Giorgia Andrisani

**Affiliations:** 1 Matera Via Della Croce 47, 75100 Matera, Italy; 2 Università Degli Studi Di Bari, Aldo Moro, Bari, Italy; 3 Ezelsveldlaan 2, 2611 rv Delft, Netherlands; 4 Universidad Alfonso X, El Sabio Villanueva de La Canada, Madrid, Spain

**Keywords:** Sleep apnea, Mesencephalic trigeminal nucleus, Ascending reticular activating system, Cyclic alternating pattern, Parabrachial nucleus

## Abstract

**Objective:**

The purpose of this study is to examine the pathophysiology underlying sleep apnea (SA).

**Background:**

We consider several critical features of SA including the roles played by the ascending reticular activating system (ARAS) that controls vegetative functions and electroencephalographic findings associated with both SA and normal sleep. We evaluate this knowledge together with our current understanding of the anatomy, histology, and physiology of the mesencephalic trigeminal nucleus (MTN) and mechanisms that contribute directly to normal and disordered sleep. MTN neurons express γ-aminobutyric acid (GABA) receptors which activate them (make chlorine come out of the cells) and that can be activated by GABA released from the hypothalamic preoptic area.

**Method:**

We reviewed the published literature focused on sleep apnea (SA) reported in Google Scholar, Scopus, and PubMed databases.

**Results:**

The MTN neurons respond to the hypothalamic GABA release by releasing glutamate that activates neurons in the ARAS. Based on these findings, we conclude that a dysfunctional MTN may be incapable of activating neurons in the ARAS, notably those in the parabrachial nucleus, and that this will ultimately lead to SA. Despite its name, obstructive sleep apnea (OSA) is not caused by an airway obstruction that prevents breathing.

**Conclusions:**

While obstruction may contribute to the overall pathology, the primary factor involved in this scenario is the lack of neurotransmitters.

## Introduction

Sleep apnea (SA) is a respiratory disorder characterized by intermittent reductions (hypopnea) or full cessation (apnea) of breathing for a few seconds or as long as a few minutes; these episodes may occur many times during sleep.^1^ More than one billion individuals worldwide currently experience some form of sleep apnea [1]. The number of individuals carrying this diagnosis has been rising steadily. SA can have a negative effect on quality of life and may also increase the risk of premature death. Of note, SA is a significant risk factor associated with mortality due to all causes, particularly those associated with cardiovascular events [2].

## Materials and methods

We reviewed the published literature focused on sleep apnea (SA) reported in Google Scholar, Scopus, and PubMed databases. We used these findings to inform our review that focused on the role of the mesencephalic trigeminal nucleus (MTN).

## Background

While several types of this disorder have been identified, the two most prominent forms are obstructive and central SA. Obstructive sleep apnea (OSA), which is the more common of the two forms, is believed to be a result of complete or partial obstruction of the upper airway during sleep. By contrast, central sleep apnea (CSA) results from dysfunction of the respiratory control centers of the brain stem, most notably the pre-Bötzinger complex. In CSA, the respiratory control center fails to provide the signal to inhale, causing the individual to miss one or more breathing cycles while asleep.

### Sleep

Sleep is a complex physiological mechanism that involves the actions of numerous components of the central nervous system (CNS). Sleep is induced and maintained by CNS-mediated inhibition of neurons in the ascending reticular activating system (ARAS) nuclei mediated by the hypothalamic neurons of the preoptic area (POA), the thalamic reticular nucleus, and the nucleus accumbens [3]. Neurons in the POA release inhibitory neurotransmitters such as ɣ-aminobutyric acid (GABA) and galanin^2^ to activate neurons in ARAS, including orexinergic neurons of the lateral hypothalamus, histaminergic neurons of the tuberomammillary nucleus, glutamatergic neurons of the parabrachial nucleus (PBN) in the dorsolateral pons, serotonergic neurons of the dorsal raphe nuclei, noradrenergic neurons of the locus coeruleus, and cholinergic neurons of the basal forebrain, laterodorsal tegmentum, and pedunculopontine tegmentum. GABA released by the hypothalamus inhibits the ARAS and neurotransmitters are not released [4, 5].

MTN consists of a narrow band of cells that passes near the periaqueductal grey and is completely incorporated in the Reticular Formation [6]. Adjacent to the MTN, towards the centre and in front of the fourth ventricle, is the locus coeruleus (LC), which is the main source of noradrenergic fibers in the CNS and is the origin of the sympathetic nervous system.

The LC and MTN are closely related during embryonic development: the LC plays a role in the differentiation of MTN neurons, which in turn are necessary for proper LC functioning (Espana A, 2012).

### The ascending reticular activating system (ARAS)

Neurotransmitters released by the ARAS activate the cerebral cortex. These neurotransmitters also control the activity of the lungs and heart [7]. Reduced levels of norepinephrine in the brainstem also contribute to the development of respiratory disturbances [8, 9]. Similarly, dopamine stimulates respiratory and cardiovascular function [10] and medullary serotonergic neurons modulate respiratory responses [11, 12]. Orexin, which is an important promoter of wakefulness, also stimulates respiratory and cardiovascular responses [13–15] as well as testosterone synthesis [16, 17]. Testosterone stimulates the activity of the respiratory and cardiovascular systems, especially in the male sex; therefore, in the male, in addition to the decreased contribution of orexin to respiratory control, it also lacks the contribution of a greater amount of testosterone. This finding may explain at least in part the prevalence of SA in men [18–22]. Likewise, neurons in the PBN play a particularly important role in SA development. These neurons respond to excitatory signals provided by the amino acid, L-glutamate as well as inhibitory signals mediated by GABA, and are modulated by signals from cholinergic, monoaminergic, and orexinergic/hypocretinergic neurons as well as those that express melanin-concentrating hormone (MCH) that are directly involved in the control of both respiratory and cardiovascular function. These distinct neuronal populations mediate continuous interactions between the cortex and subcortical circuits and can modulate sympathetic and cardiovagal output, respiratory patterns, and chemosensitivity [23, 24].

While neurotransmitter release from the ARAS remains ongoing to some extent, overproduction of GABA by the neurons in the POA during sleep might result in a significant reduction in the release of one or more of these neurotransmitters from the ARAS.

#### The mesencephalic trigeminal nucleus (MTN)

The mesencephalic trigeminal nucleus (MTN) is a brainstem structure that activates the release of neurotransmitters from the ARAS during sleep. Neurons in the MTN are activated by GABA released from the POA. Activated MTN cells respond by releasing glutamate that then activates the ARAS and promotes the release of neurotransmitters that modulate the activity of the cerebral cortex as well as the respiratory and cardiovascular systems [25]. Glutamate from MTN cells can also modulate the activity of neurons in the motor trigeminal nucleus (Mo5), thereby activating the muscles of mastication; this results in the clenching of the teeth (i.e., rhythmic masticatory muscle activity (RMMA), also known as sleep bruxism (SB)) and increasing glutamate release and activation of the ARAS [25].

#### Electroencephalographic findings during sleep

Neurons in the POA release GABA for the entire duration of sleep. Thus, the MTN must remain activated during the entire duration of sleep (most evidently, on the EEG, during NREM sleep) to maintain activation of the ARAS to support glutamate release. Of note, on an electroencephalogram (EEG), only electrocortical signals associated with responses to GABA and glutamate will be detected.

#### Electroencephalographic sleep structure

**Fig. 1:**
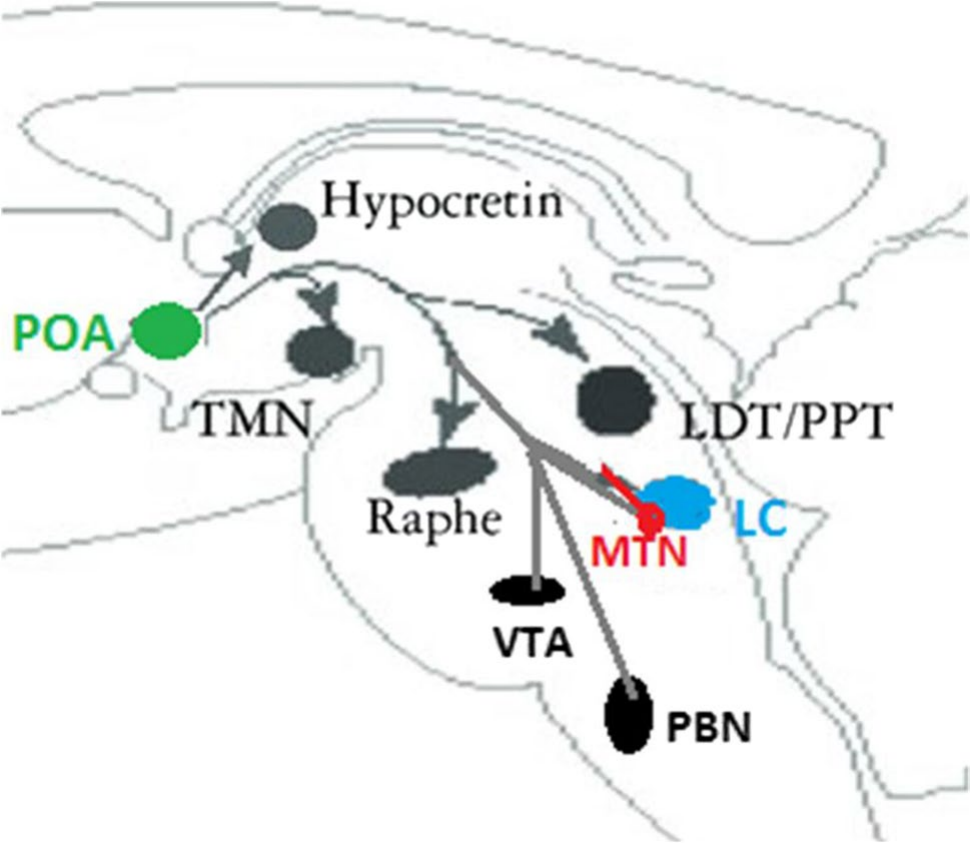
Neurons in the POA release GABA to inhibit neurons in ARAS nuclei. GABA from POA activates MTN

**Fig. 2:**
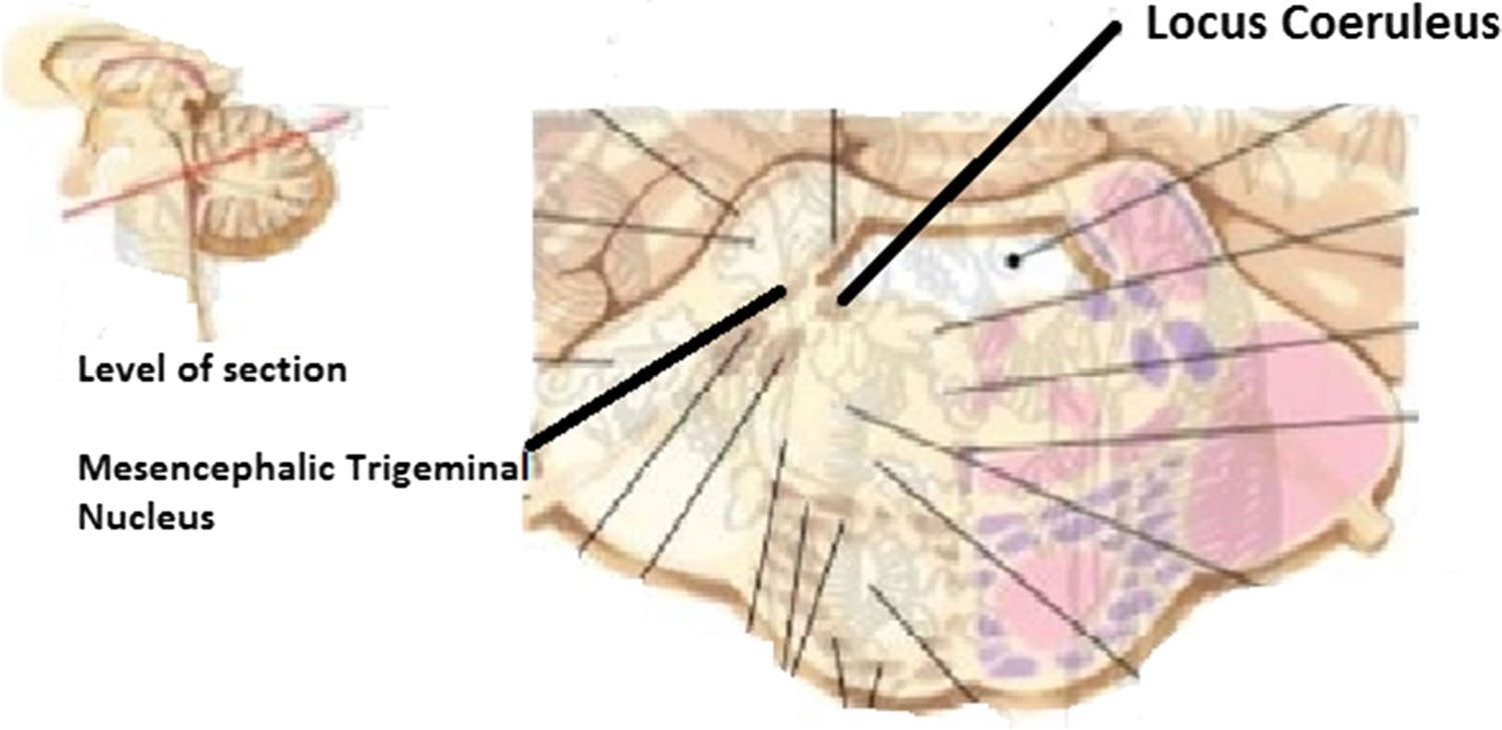
Relationship between LC and MTN

**Fig.3:**
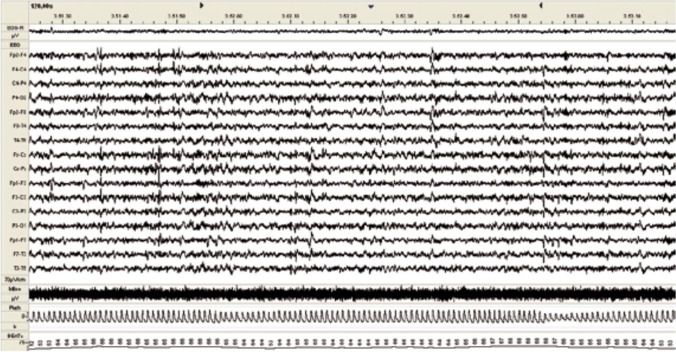
Baseline EEG tracing during NREM sleep

Sleep includes two distinct phases. The first, in which the eyes move rapidly, is known as rapid eye movement (REM) sleep. The EEG associated with this phase is rapid and unsynchronized. By contrast, the EEG pattern detected during NREM phases is slow and synchronized. An example of an NREM trace is shown in Figs. 1, 2 and 3.

**Fig. 4:**
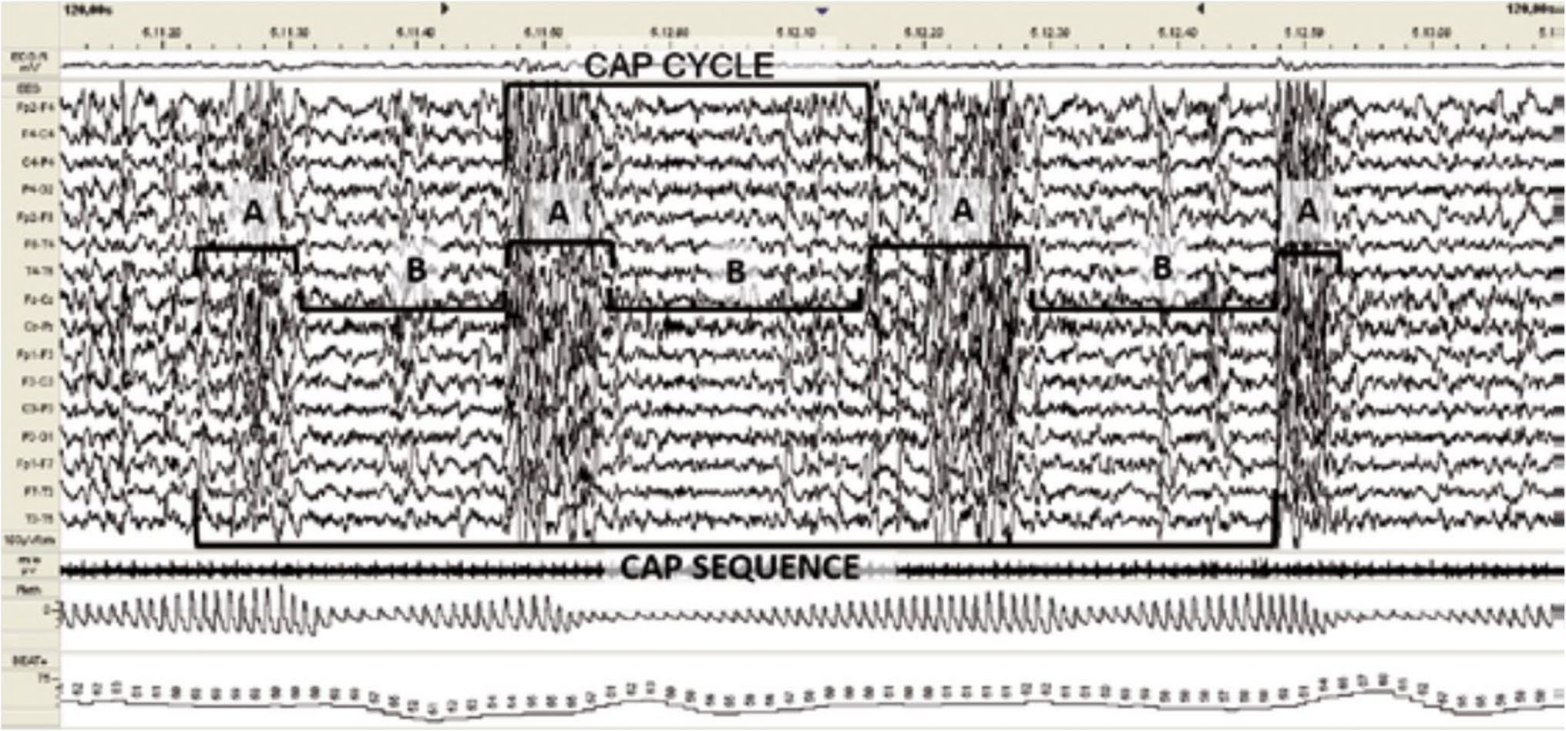
A cyclic alternating pattern (CAP) was observed on EEG during NREM sleep

In this image, we can detect both baseline traces and cycles in which these patterns have been altered. In addition to these macrostructural sleep stages, microstructural features of NREM detected on EEG include alternating fluctuations characterized as sleep “superficialization” [26]. This type of EEG trace, which is known as a “cycle alternating pattern” (CAP) can be detected for either brief or long periods and can be interrupted by periods of normal EEG activity characteristic of NREM sleep (Fig. 4).

Each CAP cycle is defined by alternating sequences of two EEG patterns known as the A and B-phases. These variations are closely related to fluctuations in the levels of arousal that characterize the two different functional states and that are mediated by the arousal control mechanism [27]. Arousal is mediated by ARAS activation [28, 29]. The A-phase represents an activation response and includes both slow high-voltage (synchronized) and fast low-voltage (unsynchronized) waves. These phases are associated with temporary increases in the level of consciousness, muscle tone, and vegetative functions associated with ARAS neuronal activation during sleep. GABA released from hypothalamic POA neurons activates the MTN which in turn activates the ARAS nuclei. Neurons in the MTN express GABA-depolarizing ionotropic receptors that produce slow but large and sustained chloride currents when activated [30, 31]. Thus, MTN neurons respond with prolonged activation that can be maintained throughout the duration of sleep. Detection of CAP and non-CAP (NCAP) phases on EEG is linked to the specific kinetics of these GABA-depolarizing receptors.

### Sleep apnea

**Fig. 5:**
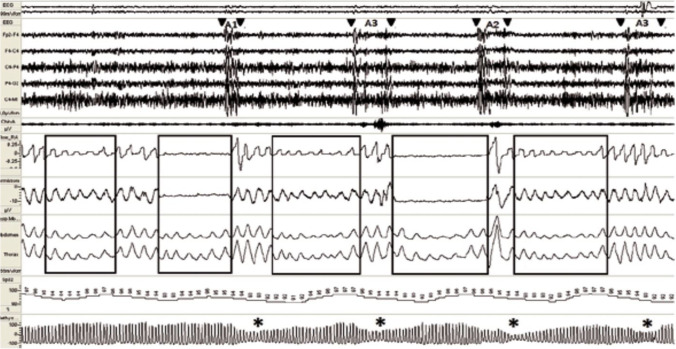
Modulation of EEG responses based on respiratory events. Respiratory events are as indicated within the boxes, including (from left to right) hypopnea, apnea, hypopnea, apnea, and hypopnea. The apneic episodes appeared exclusively during the inhibitory B-phase of NREM sleep and concluded at the beginning of the A-phase of the CAP. Resumption of breathing is always signaled by conversion to an A-phase pattern [24, 32, 33]

Apneic episodes appear often, but not always, immediately after phase A during the B-(inhibitory) phase of the CAP [24, 32, 33] (Fig. 5).

## Results (pathophysiology of sleep apnea)

Apnea occurs when brainstem structures responsible for activating the respiratory system (i.e., the ARAS nuclei and brainstem structures associated with the pre-Bötzinger complex) are inactivated (CSA) or when individuals are unable to overcome resistance (obstruction) in the airways (OSA). We hypothesize that these findings are largely the result of an excess of GABAergic-mediated inhibition of the POA and/or insufficient MTN-mediated activation of the ARAS nuclei and their collective impact on the respiratory system during sleep. In the text to follow, we analyze the various parameters revealed by EEGs performed on individuals during sleep (i.e., polysomnography (PSG)).

The only forms of cyclic activation recorded by EEG during NREM sleep were A-phases of CAPs [34, 35]. We note that only the MTN can undergo physiologic cyclic activation for the entire duration of the NREM sleep, leading to activation of the ARAS and generation of increased cortical activity recorded by the EEG; thus, we hypothesize that the CAP A-phases are regulated by signals from the MTN. We distinguish 3 types of A-Phases, A1, A2, and A3 with increasing of arousal threshold (the arousal threshold is the propensity to wake up from sleep), each A-phase marks a single cycle of neurotransmitter release from the ARAS; all A-phase subtypes were shown to be capable of reinstatement of breathing [36], with the strongest effect nonetheless noted during A3 [36, 37]. In milder obstructive sleep apnea (apnea–hypopnea index < 20), sleep continuity may be reinforced by cyclic alternating pattern subtype A1, whereas in more severe obstructive sleep apnea, decompensation of these sleep-stabilizing mechanisms may occur and more intrusive cyclic alternating pattern fluctuations disrupt sleep circuitry [37] the action of the MTN causes the release of many neurotransmitters. By contrast, the NCAP and CAP B-phases result from the actions of GABA on ARAS neurons; the MTN remains active at these times.

### Airway obstruction cannot be the sole cause of obstructive sleep apnea (OSA)

Findings from the EEG tracings reveal that apneic episodes occur exclusively during inhibitory B-phases; the resumption of breathing correlates with an activating A-phase pattern [24, 32, 33]. Apneic episodes are never detected during CAP A-phases. During this phase, neurotransmitters are released from the ARAS and apnea ceases. Of note, CAPs and apneic periods are recorded with different instruments; thus, a coincidence of events most likely represents a relationship between these two mechanisms. Of note, obstructive mechanisms modulate the responses of the respiratory system alone while central mechanisms (i.e., ARAS neurotransmitter deficiency) will have an impact on both the CNS and the respiratory system. Thus, if obstruction was the sole cause of OSA, CAP phase-B periods on EEG would be unlikely to correlate specifically with apneic events. Thus, we hypothesize that OSA may also be the result of ARAS neurotransmitter deficiency. Of note, apneic episodes are always detected in association with CAP B-phases and never during corresponding NCAP phases. Thus, SA is unlikely to be a result of GABA-mediated inhibition of the ARAS. If this was the case, SA would also be detected during NCAP phases, which are the most abundant in terms of time. By contrast, the actions of the MTN are more likely to result in apneic responses. A more careful analysis of EEG/ polysomnography (PSG) results corresponding to RMMA/SB episodes reveals that:

- RMMA episodes result from a sequence of events associated with sleep micro-arousal. During the seconds to minutes that immediately precede the onset of RMMA, the heart and brain become activated [38]; the respiration rate also increases typically before the onset of muscle activity [39]. These results imply that the MTN is active and activates the nuclei of the ARAS during the NCAP phases of sleep, thereby preventing the onset of SA. Thus, apnea can only develop once the MTN has released all its glutamate stores and fully activated the nuclei of the ARAS, leaving it functionally inactive.
- MTN remains activated throughout the duration of sleep, including during the CAP B-phases and NCAP. However, after an A-phase, which is accompanied by prominent glutamate release, the MTN may be functionally depleted, and apnea can occur. While the impact of GABA prevails during the NCAP phase, the MTN remains capable of glutamate release and apnea does not occur. Therefore, OSA is not the direct result of the airway obstruction; while this feature remains an essential co-factor in the mechanism underlying OSA, the primary contributing factor to this phenomenon is the temporary absence of ARAS-derived neurotransmitters.

### CAP rates, the PBN, and the MTN and their contributions to SA

The number of CAP events detected on an EEG during 1 h of NREM sleep is known as the “CAP-rate” (CAP rate is the percentage ratio of total CAP time to NREM sleep time [33]. This value provides us with information on MTN activity,the remainder of the EEG tracing (i.e., the NCAP) reflects GABAergic activity. Therefore, a relatively low CAP rate suggests minimal MTN activity and the predominance of GABA and its associated neurotransmitters, and vice versa [40]. Measurements of the CAP rate might provide substantial insight into the differential diagnosis of neurological pathologies and may provide a means to evaluate the effectiveness of specific pharmacological treatments.

Results from previous studies revealed that individuals manifesting symptoms of depression exhibit higher CAP rates on EEG [41]. This finding implies that their ARAS nuclei are comparatively inactive and release lower levels of neurotransmitters in the CNS (i.e., less GABA leads to a higher CAP rate). This information might ultimately be developed and used to confirm the diagnosis of depression and to determine whether the prescribed therapies are having an impact at least at the physiological/neurologic level.

Similarly, patients diagnosed with migraine were found to have lower than normal CAP rates [42]. This information, which reflects an overabundance of neurotransmitters, might also contribute to the differential diagnosis of this neurological condition.

Given that mechanisms underlying SA include excess GABA release by the POA, the CAP rate detected on EEG should be at normal or lower than normal levels [43]. However, higher than normal CAP rates were detected in patients diagnosed with severe SA [33]. In general, patients with severe SA (i.e., those with apnea/hypopnea index (AHIs) ≥ 20 events/h) exhibit higher CAP rates on EEG; by contrast, those with mild to moderate disease (AHI < 20 events/h) have normal or reduced CAP rates [43]. Among the explanations for these observations, Kaur et al. [44] described several subcortical arousal circuitries and hypothesized that overly intense physiological stimulation (i.e., AHI ≥ 20events/h) may overwhelm the brain’s sleep-gating mechanisms. Among their findings, Kaur et al. [44] demonstrated the existence of ascending and descending projections from subregions of PBN that play a central role in inducing cortical arousal and regulating respiratory responses. Their results also revealed two functionally distinct arousal states that potentially arise from the ascending projections of the PBN with links to the basal forebrain and other hypothalamic, insular, and thalamo-cortical targets. In this model, baseline arousal is supported, at least in part, by excitatory inputs from the medial PBN. This state is altered in response to sleep-active GABAergic neurons in the medullary parafacial zone; this leads to profound increases in the synchronization of slow-wave sleep [45]. By contrast, visceral sensory distress (e.g., pain or respiratory insufficiency) stimulates the lateral subregions of the PBN and evokes more potent basal forebrain arousal [44]. The electrocortical signature of this activity includes higher alpha/beta frequency bursts that are consistent with the A-phase of the CAP response. Furthermore, in experiments performed in rodents, hypercapnic stimulation of the lateral PBN in rodents led to a powerful concomitant resumption of breathing via its descending medullary projections [44, 46]. This property of the PBN is somewhat reminiscent of the dual activation capacity of the MTN and its role in promoting motor (Mo5) activation that leads to RMMA and SB [25, 38, 39, 47–51]. Activation of the MTN in this setting leads to the release of larger quantities of glutamate and thus increased activation of ARAS neurons, particularly those of the PBN that lead to increases in heart, respiratory, and CAP rates. Of note, excess GABA released by the POA is the source of “visceral distress” that leads to the activation of the MTN. The fact that ARAS neurons (the PBN in particular) and MTN exhibit two functionally distinct arousal states is unlikely to be a coincidence [52].

### Sleep apnea (SA) and REM sleep

As noted above, CAPs are detected only during periods of NREM sleep. Nonetheless, SA can also occur during REM sleep. This is because ARAS neurotransmitters may be released in reduced quantities or not released at all. This will prevent the switch from NREM to REM sleep cycles. Osorio-Forero et al. [53] reported that norepinephrine levels declined steeply during REM sleep. Released neurotransmitter from locus coeruleus REM-OFF neurons acts on the pedunculopontine tegmentum REM-ON neurons to prevent REM sleep; thus, norepinephrine levels tend to be very low during this sleep cycle, and SA can develop. Likewise, the orexinergic tone is completely absent during REM sleep [54]. Both dorsal raphe nucleus serotonergic and TMN histaminergic neurons appear to be electrically silent during REM sleep [55, 56]. Therefore, ARAS neurotransmitter deficiencies associated with SA also persist during REM sleep.

### Causes of sleep apnea (SA)

The absence of one or more neurotransmitters during sleep may be due to the excessive actions of the POA (i.e., GABA release), but may also be due to an ineffective MTN. Among the mechanisms associated with an ineffective MTN, we consider the following:

- Sudden infant death syndrome (SIDS) can occur in newborns, mainly between the second and sixth months of life. During the first month of life, a newborn does not experience true NREM but instead exhibits a unique type of REM sleep. On or about the sixth month of life, deciduous teeth emerge and the MTN becomes more effective [57]. Results from multiple studies document that pacifier use can reduce the risk of SIDS [58], and can also activate the MTN.
- The MTN may also be rendered ineffective in elderly, edentulous individuals who need to sleep with their mouths open in order to breathe because of airway obstruction [59–62].

### Diagnosis

A diagnosis of SA can be established by the results of a PSG examination or (more easily but less precisely) via the use of an oximeter. Many patients are diagnosed based on reports from spouses that he or she stops breathing for a substantial period of time while asleep. Even if the diagnosis is clear, it is important to understand why and how this patient developed SA. This will require a careful medical history and clinical examination to detect problems that may include substance abuse, drugs, obstructions, and lack of or complete absence of teeth. It is not possible to treat SA without identifying and (if possible) eliminating the underlying cause.

### Agents that can cause sleep apnea

Substances and drugs that over-stimulate GABA release from the POA can cause or exacerbate SA. Among the risk factors in this category, attention should be paid to alcohol [63] and tobacco use [64], use of opioids and/or cannabis [65, 66], and obesity [67]. Several of these agents may also result in SB [68–71]. Furthermore, some anticonvulsants that amplify signaling via GABA-A receptors can elicit or exacerbate SA [72]. Similarly, some antidepressants increase the levels of neurotransmitters detected in the CNS [73]. The use of some anticonvulsant [70] and antidepressant medications [74] can also result in SB. Of note, none of these drugs promote airway obstruction.

## Therapies for sleep apneas

Some alternative antidepressants that might be considered include the selective norepinephrine reuptake inhibitor, reboxetine together with the antimuscarinic hyoscine butylbromide [75], and the serotonin modulator, trazodone [76]; mirtazapine is another potential choice [77]. Patients often receive supplemental oxygen administered via C-PAP and BiPAP, among other devices. While these therapies provide excellent support for increased oxygen intake of oxygen, they may not be a very effective means to treat cardiac complications associated with central apneas. Many of these same antidepressants can also be used to treat SB (Grinshpoon A, 2014; Khosravi M, 2020).

The use of the anti-hypertensive agent, clonidine, has been associated with diminished susceptibility to central apnea [78] and can also be used to treat SB [79, 80]. Interestingly, clonidine is a selective centrally-acting alpha-2-adrenergic agonist that alters the NREM/REM cycle via dose-dependent reductions in REM sleep [81]. Clonidine can be used to treat SA and SB and also reduces the incidence of RMMA while increasing the CAP rate [82]. While this appears to be a paradoxical effect of this drug, it is important to recognize that although Mo5 contains many adrenergic receptors [83, 84], clonidine activates only those of the α2 class. Hence, clonidine inhibits Mo5 while stimulating GABA release in the hypothalamus and thus activating MTN neurons [85–87]. Thus, clonidine appears to be an excellent drug that might be used for the management of SA. Further understanding of the impact of clonidine on Mo5 and MTN will require additional research.

As a final note, we ask our readers to understand that this chapter was not designed to be an exhaustive survey of drugs that might cause or be used to treat SA. Instead, we have chosen to highlight some of the agents that can cause SA and discuss some of the drugs that might be used for its treatment.

## Sleep apnea and sudden unexplained death during sleep (SUDS)

SA and its associated causes are quite hazardous to one’s health, most notably in patients with pre-existing cardiac and bronchopulmonary pathology. Three hundred to four hundred thousand of these patients die during sleep each year in the USA alone [2, 88–91]. We have already discussed SIDS and its impact on infants. While the pathogenesis of SUDS has not yet been elucidated, families with a significant history of this disorder are more likely to present with significant nocturnal hypoxia [92–94] and a lack of ARAS neurotransmitters [95].

## Discussion

MTN is the only structure in the brainstem (and perhaps in the entire CNS) that remains activated during sleep. The MTN can activate nuclei of the ARAS followed by the cerebral cortex as well as the cardiovascular and respiratory systems. These findings were confirmed in PSG studies in which different instruments (e.g., EEG, ECG, EMG, pulse oximeter, and nasal cannulas) simultaneously record the results of MTN activation that correspond to episodes of SA and RMMA. EEGs performed during sleep permit us to distinguish between different phases of CAP and NCAP; these tracing also distinguish between phases of greater (CAP phase-A) and reduced cerebral activation (CAP phase-B). Notably, SA occurs only during intervals corresponding to CAP phase-B. Because CAP phase-A corresponds to activation of the MTN and NCAP phases feature increases in heart and respiratory rate, these results suggest SA occurs only when the MTN has become nearly inactive. These features are also observed in OSA; thus, the key element of this response appears to be MTN inactivity. This is especially the case in “fragile” patients, including those with respiratory problems.

One key finding of our study was that the main cause of OSA cannot be obstruction per se. If this was the case, then episodes of SA should also be prominent during the NCAP phases. While the pathogenetic mechanisms we have described are clearly valid for CSA (i.e., decreased release of ARAS neurotransmitters prevents the appropriate functioning of the respiratory system both directly and via mechanisms promoting dysfunction of the parabrachial nucleus and pre-Bötzinger complex), they also explain many of the signs and symptoms of more commonly diagnosed OSA. As a case in point, we considered what might happen in response to an MTN that has not been fully activated (e.g., in the absence of teeth in both newborns and the elderly). In both cases, our findings revealed an increase in the number of unexplained deaths during sleep, suggesting that the pathogenic mechanisms underlying both SIDS and SUDS may relate directly to the absence of ARAS-derived neurotransmitters and SA.

We would like to point out that the apneas we talked about in this paper, sleep apneas, occur almost exclusively in healthy individuals or with small comorbidities (obstructions, obesity) and that, in these patients, apneas never occur during wakefulness, therefore sleep, with its mechanisms, is a “conditio sine qua non” for apneas. There are other apneas such as those occurring in Cheyne–Stokes breathing, in which the apnea can also occur while awake, but we are talking about patients with major problems: damage to respiratory centers, or by heart failure, newborns with immature respiratory systems, kidney failure, narcotic poisoning, and raised intracranial pressure. The pathophysiology of Cheyne–Stokes breathing can be summarized as apnea leading to increased CO_2_ which causes excessive compensatory hyperventilation, in turn causing decreased CO_2_ which causes apnea, restarting the cycle, it is not related to sleep, but can also occur during sleep.

Precisely the fact that apneas have a strong correlation with the B-phase of the CAPs (when the action of the MTN has finished) and not in the A-phase or in the NCAP phases, when the MTN is active, perhaps less active than POA GABA, but nonetheless active, provides evidence that apneas are due to one or more ARAS neurotransmitters deficiency.

## Conclusions and suggestions

CAPs identified on EEGs are generated in response to the activation of the MTN.

SA is the result of a deficiency of one or more neurotransmitters released by the ARAS. This deficiency has been reported in association with both REM and NREM sleep. Therefore, SA can occur during both phases.

While deficiencies in one or more of the ARAS neurotransmitters during sleep may be due to the excessive activation of the POA (i.e., the release of GABA), this state may also be due to MTN in an ineffective state.

Despite its name, OSA is not caused by an airway obstruction that prevents breathing. While obstruction may contribute to the overall pathology, the primary factor involved in this scenario is the lack of neurotransmitters.

SA and the associated deficiency of ARAS-derived neurotransmitters have been linked to unexplained death during sleep. We recognize that these neurotransmitters also act on the cardiovascular system. Thus, the overall lack of neurotransmitters may also lead to cardiovascular problems, including cardiac arrest and death.

SB results in an increase in the level of ARAS neurotransmitter release and by doing so prevents the development of SA and associated heart problems. Patients presenting with signs and symptoms of SB might be responding by activating a defense mechanism against GABA excess.

Substances and drugs that over stimulate the release of GABA from the POA can cause or exacerbate pre-existing SA.

In conclusion, the pathophysiologic factors associated with the development of SA are directly related to central mechanisms modulating normal sleep. Patients presenting with suspected SA might be managed with an oximeter and heart rate monitor equipped with a powerful alarm. Thus, if the oxygen saturation and/or heart rate drop too low, an alarm will be activated. This very simple strategy may ultimately save his or her life. Devices of this type are already available for purchase; they are inexpensive and may save many lives if properly calibrated and used on a routine basis.

## Data Availability

I confirm I have included a data availability statement in my main manuscript file.

## Footnotes

1 For simplicity, this review will focus primarily on SA, but will also refer to sleep hypopnea as relevant.

2 In the text to follow, we refer to GABA only.

## Abbreviations

AHI: Apnea hypopnea index
ARAS: Ascending reticular activating system
BiPAP: Bilevel positive Airway pressure
CAP: Cycle alternating pattern
CNS: Central nervous system
SA: Sleep apnea
CPAP: Continuous Positive Airway Pressure
CSA: Central sleep apnea
GABA: ɣ-Aminobutyric acid
ECG: Electrocardiogram
EEG: Electroencephalogram
EMG: Electromyogram
LC: Locus coeruleus
MCH: Melanin-concentrating hormone
Mo5: Motor trigeminal nucleus
MTN: Mesencephalic trigeminal nucleus
NCAP: Non cycle alternating pattern
NREM: Non rapid eye movement
OSA: Obstructive sleep apnea
PBN: Parabrachial nucleus
POA: Preoptic area
PSG: Polysomnography
REM: Rapid eye movement
RMMA: Rhythmic masticatory muscle activity
SB: Sleep bruxism
SIDS: Sudden infant death syndrome
SUDS: Sudden unexplained death syndrome
TMN: Tubero-mammilary nucleus
